# Testosterone Decreases Placental Mitochondrial Content and Cellular Bioenergetics

**DOI:** 10.3390/biology9070176

**Published:** 2020-07-20

**Authors:** Jay S. Mishra, Chellakkan S. Blesson, Sathish Kumar

**Affiliations:** 1 Department of Comparative Biosciences, School of Veterinary Medicine, University of Wisconsin, Madison, WI 53706, USA; jay.mishra@wisc.edu; 2 Reproductive Endocrinology and Infertility Division, Department of Obstetrics and Gynecology, Baylor College of Medicine and Family Fertility Center, Texas Children’s Hospital, Houston, TX 77030, USA; selvanes@bcm.edu; 3 Department of Obstetrics and Gynecology, School of Medicine and Public Health, University of Wisconsin, Madison, WI 53792, USA; 4 Endocrinology-Reproductive Physiology Program, University of Wisconsin, Madison, WI 53715, USA

**Keywords:** testosterone, preeclampsia, mitochondria, placenta, trophoblast, respiration, oxygen consumption, PGC-1α, NRF1

## Abstract

Placental mitochondrial dysfunction plays a central role in the pathogenesis of preeclampsia. Since preeclampsia is a hyperandrogenic state, we hypothesized that elevated maternal testosterone levels induce damage to placental mitochondria and decrease bioenergetic profiles. To test this hypothesis, pregnant Sprague–Dawley rats were injected with vehicle or testosterone propionate (0.5 mg/kg/day) from gestation day (GD) 15 to 19. On GD20, the placentas were isolated to assess mitochondrial structure, copy number, ATP/ADP ratio, and biogenesis (Pgc-1α and Nrf1). In addition, in vitro cultures of human trophoblasts (HTR-8/SVneo) were treated with dihydrotestosterone (0.3, 1.0, and 3.0 nM), and bioenergetic profiles using seahorse analyzer were assessed. Testosterone exposure in pregnant rats led to a 2-fold increase in plasma testosterone levels with an associated decrease in placental and fetal weights compared with controls. Elevated maternal testosterone levels induced structural damage to the placental mitochondria and decreased mitochondrial copy number. The ATP/ADP ratio was reduced with a parallel decrease in the mRNA and protein expression of Pgc-1α and Nrf1 in the placenta of testosterone-treated rats compared with controls. In cultured trophoblasts, dihydrotestosterone decreased the mitochondrial copy number and reduced PGC-1α, NRF1 mRNA, and protein levels without altering the expression of mitochondrial fission/fusion genes. Dihydrotestosterone exposure induced significant mitochondrial energy deficits with a dose-dependent decrease in basal respiration, ATP-linked respiration, maximal respiration, and spare respiratory capacity. In summary, our study suggests that the placental mitochondrial dysfunction induced by elevated maternal testosterone might be a potential mechanism linking preeclampsia to feto-placental growth restriction.

## 1. Introduction

Preeclampsia (PE) is a severe pregnancy-specific multi-system disorder characterized by the new onset of hypertension, proteinuria, edema, and a series of other systematic dysfunctions after 20 weeks of gestation [1,2]. PE affects about 5% of all pregnancies worldwide and remains the major cause of maternal and fetal death [2,3,4]. Despite increased efforts, the pathophysiological mechanisms of PE remain unclear. However, the placenta is implicated to play a crucial role in the progression of this disease [5].

The placenta is a highly metabolically active organ with an active role in transporting nutrients, as well as modifying the composition of some nutrients through its metabolic activity [6]. Mitochondria are the main energy producers in the cell. They consume oxygen and produce ATP by electron transport and oxidative phosphorylation. The metabolic activity of the placenta is sustained throughout gestation by increasing mitochondrial activity and biogenesis [7]. There is evidence that the dysfunction of the placental mitochondria with an excessive generation of reactive oxygen and nitrogen species is observed in the PE placenta [8,9]. In addition, the mitochondrial structure was found to be disorganized with the disappearance of cristae in trophoblast cells of PE placenta [10,11]. In addition to the structural damage, placental mitochondrial electron chain enzymes are also inhibited in PE [12]. In fact, the incidence of PE is higher in the family with mitochondrial dysfunction [13]. Together, these findings suggest that mitochondrial function plays an important role in the pathogenesis of PE. Nevertheless, the underlying etiological factor that induces perturbations in the mitochondrial function in the placenta has not been established. The finding that endothelial cells incubated with plasma from PE women result in a significant decrease in mitochondrial function [14] suggests that circulatory factors/hormones have the ability to impair mitochondrial bioenergetics.

Several lines of evidence indicate that the plasma levels of testosterone (T) are 2-fold higher in PE pregnancies compared to normal pregnancies [15]. Higher maternal T levels have long been associated with adverse pregnancy outcomes, both for the mother and her fetus. T increase in early pregnancies could impair trophoblast invasion [16] and placental differentiation [17]. However, the majority of the studies, i.e., 12 out of the 14 epidemiological studies, show that T increase in PE pregnancies occurs later in gestation (around 28 to 38 weeks). Experimental studies mimicking such an increase in T levels later in gestation are shown to exhibit many characteristic PE manifestations. The T increase in the later part of rat pregnancy has been shown to exhibit endothelial dysfunction and increase blood pressure [18,19], increase vascular sensitivity to angiotensin II [18,20], decrease spiral artery elongation and placental vascularization [21], and reduce placental nutrient transport capacity [22]. Additionally, elevated T during pregnancy is associated with decreased fetal growth and increased risk for adult-onset diseases [23,24]. Elevated maternal T levels can impact fetal growth through at least two potential effects. Studies have demonstrated that maternal T can cross the placenta and directly cause fetal damage [25,26,27]. On the other hand, T can also affect fetal growth due to its indirect action on the placenta. It has been shown that rat placenta and human trophoblast cells express androgen receptor (AR) [28,29,30], and elevated T causes significant increases in oxidative stress in the placenta [31]. However, whether the elevated T present in PE patients contributes to the placental mitochondrial dysfunction is not known.

In normal cells, the transcriptional coactivator peroxisome proliferator-activated receptor gamma co-activator 1-alpha (PGC-1α) interacts with multiple transcription factors to coordinate energy metabolism and mitochondrial biogenesis [32]. PGC-1α primarily regulates mitochondrial biogenesis through the regulation of Nuclear Respiratory Factor 1 (NRF1) [33], which in turn plays a critical role in maintaining the copy number and structure of mitochondrial DNA [34,35]. Thus, PGC-1α and NRF1 have an important role in maintaining normal placental function. However, the response of placental PGC-1α and NRF1 expression to elevated T is not known.

In this study, we hypothesized that elevated maternal T induces damage to placental mitochondria and decreases bioenergetic profiles with an associated decrease in PGC-1α and NRF1 expression. To test this hypothesis, we mimicked a 2-fold increase in T levels in pregnant rats and examined (1) whether the structure and number of mitochondria are altered in the placenta of T-treated pregnant rats; (2) whether the amount of placental metabolic activity is decreased in the placenta of T-treated pregnant rats; and (3) whether the expression of Pgc-1α and Nrf1, the two genes important in regulating mitochondrial biogenesis and replication is altered in the placenta of T-treated pregnant rats. Finally, we also examined whether the mitochondrial oxygen consumption and bioenergetics are perturbed in in vitro cultured trophoblasts exposed to T.

## 2. Materials and Methods

### 2.1. Animals

All protocols were carried out as per National Institutes of Health guidelines (NIH Publication No. 85–23, revised 1996) with approval by the Institutional Animal Care and Use Committee at the University of Wisconsin at Madison (IACUC protocol V005847). Timed-pregnant Sprague–Dawley rats were purchased from Envigo Laboratories (Indianapolis, IN) and were maintained on 12L/12D cycles in a temperature-controlled room (23 °C) and provided with food and water *ad libitum*. On Day 15 of pregnancy, rats were divided into control and treatment groups. The control group received sesame oil (vehicle; *n* = 6) subcutaneously, and the treatment group received T propionate (Sigma, St. Louis, MO, USA) (0.5 mg/kg; *n* = 6) subcutaneously from day 15 to 19 of gestation, as previously described [18,19]. This dose and duration of T propionate were selected to mimic the pattern and increases in T levels as in PE pregnancies [20,21,22]. Rats were sacrificed at gestational day 20 by CO_2_ asphyxiation, and maternal blood was collected and centrifuged, and the plasma was stored at −80 °C for later measurement of T levels. Laparotomy was performed, and the fetuses and placentas were collected and quickly dried on blotting paper to remove any remaining fetal membranes and counted and weighed. The placentas in each litter were pooled and cut into smaller pieces and stored at −80 °C for subsequent gene and protein expression analysis.

### 2.2. Plasma T levels

T levels were measured using an ELISA kit (RTC001R; BioVendor, Asheville, NC, USA) as per the manufacturer’s instructions. The minimum detectable concentration of T is 6 pg/mL, and the intra- and inter-assay coefficients of variation for T assay were lower than 8%.

### 2.3. Electron Microscopy

Placental samples for transmission electron microscopy were fixed in 2% glutaraldehyde, and secondary fixation was achieved with osmium tetroxide. Samples were sequentially dehydrated with increasing concentrations of ethanol and embedded in an epoxy resin [36]. Cut sections were stained with uranyl acetate and lead citrate and were visualized using a 1001Hitachi H-7500 transmission electron microscope (Jeol Hitachi High-Technologies CorporationLtd., Tokyo, Japan). Mitochondrial ultrastructure was evaluated by three objective criteria by an experienced investigator in a blinded fashion. They were considered having normal or abnormal ultrastructure based on (1) the mitochondrial overall shape and structure, (2) outer and inner membrane integrity and (3) organization of the cristae. At least two out of three criteria should be met to consider mitochondria normal or abnormal. TEM images were observed at 8000×, and morphologically normal and abnormal mitochondria were counted and expressed as a percentage for each field. The percentage of morphologically abnormal mitochondria were quantitated by examining 10 fields per section.

### 2.4. Mitochondrial DNA Copy Number

Mitochondrial DNA copy number was quantified by the real-time-PCR based method using a mitochondrial DNA copy number assay kit (MCN2; Detroit R&D, Detroit, MI, USA) as per the manufacturer’s instructions [37,38]. Reactions were performed with 10 ng of DNA, and mitochondrial DNA copy numbers were normalized with nuclear DNA copy number using the 2^–ΔΔCT^ method.

### 2.5. ATP/ADP Ratio

Intracellular ATP to ADP ratio in placental tissue was quantified using the ApoSENSOR ADP/ATP kit (K225; Biovision, Milpitas, CA, USA) as described previously [37,39]. Briefly, 100 μL reaction buffer containing ATP monitoring enzyme and nucleotide releasing buffer were used as blank to determine the background luminescence. Then, 20 mg placental lysate was treated with the nucleotide-releasing buffer for 5 min. ATP levels were assessed by the addition of 1 µL of the ATP monitoring enzyme followed by the immediate measure of ATP content by using a luminometer. After 10 min, 1 µL of ADP converting enzyme was added to measure the ADP content. Based on these values, the ATP/ADP ratio was calculated.

### 2.6. Quantitative Real-Time (qRT)-PCR

Total RNA was extracted using the RNeasy mini kit (QIAGEN, Valencia, CA, USA) according to the manufacturer’s instructions. The concentration of RNA and its integrity were determined using a DS-11 spectrophotometer (DeNovix, Wilmington, DE, USA). Total RNA (1 µg) was reverse transcribed using an iScript cDNA synthesis kit (Bio-Rad, Hercules, CA, USA). After dilution, cDNA corresponding to 100 ng of RNA was amplified by qRT-PCR using a CF × 96 real-time thermal cycler (Bio-Rad, Hercules, CA, USA). Gene-specific primers were designed for Pgc-1α and Nrf1 purchased from Integrated DNA Technologies (Coralville, IA, USA). The primer sequence is provided in Table 1. Results were calculated using the 2^–ΔΔCT^ method and expressed in fold change of the gene of interest in treated versus control samples. PCR efficiencies were determined as described by Svec et al. [40]. All primers exhibited efficiency between 95% and 101%. All reactions were performed in duplicate, and β-actin was used as an internal control.

### 2.7. Western Blotting

Rat placental tissues were homogenized in ice-cold radioimmunoprecipitation assay (RIPA) buffer (Cell Signaling Technology, Danvers, MA, USA) containing a protease inhibitor tablet and phosphatase inhibitor cocktail-2 and -3 (Sigma) and kept on ice for 20–30 min with intermittent tapping for proper lysis. Lysates were cleared by centrifugation at 14,000× *g* for 10 min at 4 °C. Protein concentration was determined by a BCA assay kit (Pierce; Thermo Scientific, Waltham, MA, USA). Loading samples were prepared by mixing 40 μg proteins with NuPAGE lithium dodecyl sulfate sample buffer and reducing agent (Invitrogen; Thermo Scientific, Waltham, MA, USA) and resolved on 4%–12% gradient NuPAGE Bis-Tris gels (Invitrogen; Thermo Scientific, Waltham, MA, USA) at 100 V for 2–3 h at room temperature alongside negative control and Precision Plus Standard (Kaleidoscope; Bio-Rad, Hercules, CA, USA). After separation on the gel, proteins were transferred onto Immobilon-P membranes (Millipore, Billerica, MA, USA) at 100 V for 2 h. The membrane was blocked with 5% (*wt*/*vol*) nonfat dried milk for 1 h at room temperature. Blots were incubated overnight at 4 °C with respective primary antibodies against Pgc-1α (ab191838; Abcam, Cambridge, MA, USA), Nrf-1 (ab175932; Abcam, Cambridge, MA, USA) and β-actin (Cell Signaling Technologies, Danvers, MA, USA). After washing, the membranes were incubated with horseradish peroxidase (HRP)-conjugated secondary antibodies for one hour and then developed using the Pierce ECL detection kits (Thermo Scientific, Waltham, MA, USA). The densitometric analysis was done using Image J software. Results are expressed as ratios of the intensity of a specific band to that of β-actin.

### 2.8. Cell culture and Cell-Based Assays

Human placental trophoblast (HTR-8/SVneo) cells were obtained from American Type Cell Culture (ATCC, Manassas, VA, USA). Cells were cultured in phenol red-free RPMI 1640 (Gibco; Thermo Scientific, Grand Island, NY, USA) containing 5.5 mmol/L glucose, 10% charcoal-stripped fetal bovine serum (FBS) (Gibco), 100 units/mL penicillin, and 100 μg/mL streptomycin (Gibco). Cells were plated at a density of 0.5 × 10^6^ cells/well on 6-well plates and cultured for 24 h. Then, cells were treated with dihydrotestosterone (DHT, 0.3, 1, and 3 nM) (Sigma) for another 24 h. An indicator of cell damage, the concentrations of lactate dehydrogenase (LDH) were analyzed using a commercially available kit as per manufacturer’s instructions (Thermo Fisher Scientific, Waltham, MA, USA). The control and DHT-treated cells were processed to assess mitochondrial DNA copy number and mRNA expression of PGC-1α, NRF1, FIS-1, DRP-1, MFN-1, MFN-2, and OPA-1 (primers listed in Table 1), as described earlier.

### 2.9. Mitochondrial Oxygen Consumption

Mitochondrial bioenergetics was assessed using an XF96e Extracellular Flux Analyzer and Seahorse XF Cell Mito Stress Test Kit (Agilent Seahorse, Billerica, MA, USA), as described previously [41,42]. The trophoblasts were seeded in V7 Seahorse micro-well plates at 3.5–4.0 × 10^4^ cells/well in 100 μL standard growth media. Cells were treated with DHT and incubated at 37 °C and 5% CO_2_ for 24 h. Following treatments, culture media was changed to a non-buffered DMEM media, to allow temperature and pH equilibrium. Initially, oxygen consumption rates (OCR) were measured simultaneously three times to establish a baseline rate. Then, to evaluate the mitochondrial function, oligomycin (1 µM), carbonyl cyanide 4-(trifluoromethoxy)phenylhydrazone (FCCP, 0.5 µM), and a mixture of rotenone and antimycin A (Rot/AntA, 0.5 µM) were injected into each well sequentially, with intervals of 3–5 min of mixing between the injections, to respectively inhibit the ATP synthase, uncouple oxidative phosphorylation, and inhibit mitochondrial respiration. OCR measurements were performed before and after each addition of the given compounds. Six mitochondrial respiration parameters were determined: basal, ATP production-linked, maximal, proton leak-linked OCR, and spare respiratory capacity. OCR measurements were normalized to protein content using the BCA method.

### 2.10. Statistical Analysis

Statistical analyses were performed using GraphPad Prism software. Data are presented as the mean ± SEM. Comparisons between the two groups were performed using unpaired Student t-tests. Comparisons between multiple groups were performed using ANOVA, followed by Dunnett’s post hoc analysis. Differences were considered to be statistically significant at *p <* 0.05.

## 3. Results

### 3.1. Placental and Fetal Weights

**Figure 1:**
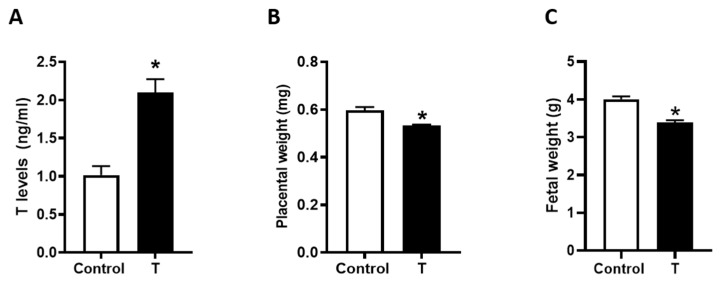
Plasma T levels and placental and fetal weights in control and T-treated pregnant rats. Pregnant rats were treated with vehicle (sesame oil) or T propionate from gestation day 15 to 19 and euthanized on day 20. (**A**) Plasma T levels were quantified using ELISA. (**B**) Placental and (**C**) Fetal weights were measured. Data presented as mean ± SEM of 6 rats in each group. * *p <* 0.05 vs. control.

Administration of T to pregnant rats from GD 15 to 19 produced a 2-fold increase in maternal circulating T levels (2.1 ± 0.17 versus 1.0 ± 0.12 ng/mL) (Figure 1A). Compared to controls, T treatment significantly decreased placental weights by 11% (0.59 ± 0.14 g versus 0.53 ± 0.33 g, Figure 1B) and fetal weights by 15% (4.0 ± 0.07 g versus 3.4 ± 0.06 g, Figure 1C). T exposure did not affect litter size (control: 12.1 ± 0.46 and T: 12.2 ± 0.52) and sex ratio (percent of males per litter control: 47.0 ± 3.95% and T: 50.4 ± 4.24%).

### 3.2. Placental Mitochondrial Ultrastructure

**Figure 2:**
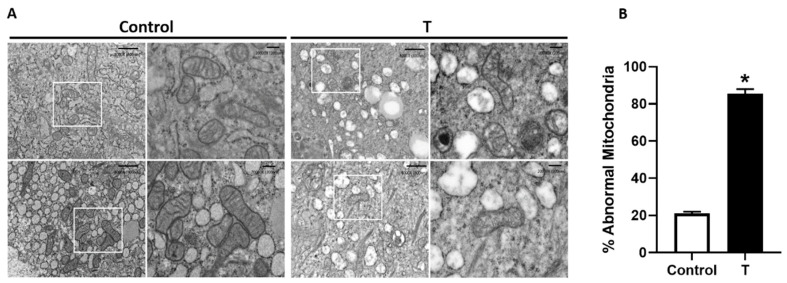
Characterization of mitochondrial structure in the placenta. (**A**) Representative electron micrographs of placental mitochondria from control (left) and T-treated pregnant rats (right). Images show less abundant mitochondria and abnormal mitochondrial structure with condensed matrix and cristae in the placenta of T-treated rats. (**B**) Quantification of the percentage of morphologically abnormal mitochondrial showing condensed matrix and cristae in the placenta of control and T-treated rats. *n* = 4 in each group. * *p <* 0.05 vs. control.

Figure 2 shows the high-magnification electron microscopic images of mitochondria from control and T placenta. The left image showed a clearly defined electron-dense matrix and cristae with intact mitochondrial membranes in the control placenta. However, the mitochondria of the T placenta were less abundant, and appearance showed degenerative changes with condensed cristae and matrix (Figure 2A). Importantly, more than 80% of the mitochondria were abnormal in the placenta of T-treated rats compared to 20% abnormal mitochondrial in the controls (Figure 2B).

### 3.3. Placental Mitochondrial Content and Metabolic Activity

**Figure 3:**
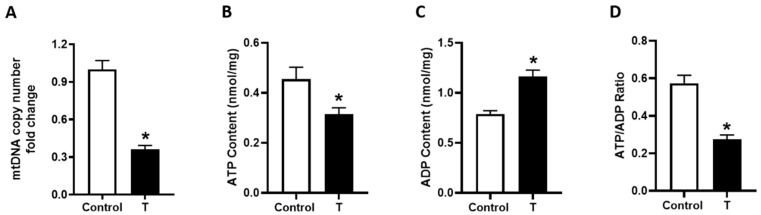
Mitochondrial copy number and ATP levels in the placenta of control and T-treated pregnant rats. (**A**) Mitochondrial DNA copy number was quantified using qRT-PCR based analysis. Placental (**B**) ATP and (**C**) ADP content was quantified using ApoSENSOR ADP/ATP kit. (**D**) Measurement of the ATP/ADP ratio. Data presented as mean ± SEM of 6 rats in each group. * *p* < 0.05 vs. control.

To evaluate the role of elevated T in mitochondrial content, we measured mitochondrial DNA copy number. Elevated maternal T levels caused a 64% reduction in mitochondrial DNA copy number compared with controls (Figure 3A). We also investigated mitochondrial metabolic activity by measuring total ATP and ADP concentration. The amount of placental ATP was decreased by 31% in the T placenta (*p <* 0.05; Figure 3B). In contrast, ADP content was increased (*p* < 0.05), and the ATP/ADP ratio was reduced drastically (−52%) in T placenta (*p* < 0.05; Figure 3C,D).

### 3.4. Pgc1-α and Nrf1 Expression in Placenta

**Figure 4:**
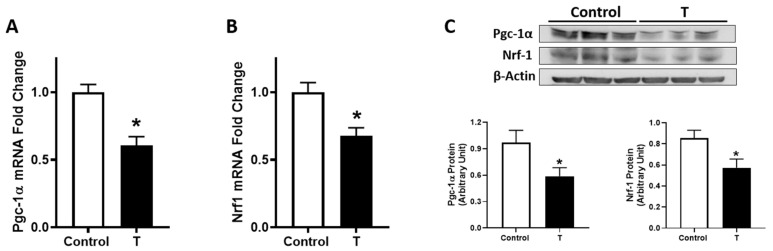
Expression of mitochondrial biogenesis indicators in the placenta of control and T-treated pregnant rats. Real-time PCR was used to assess (**A**) Pgc-1a and (**B**) Nrf1 mRNA expression in the placenta. Quantitation of placental Pgc-1a and Nrf1 mRNA expression was normalized relative to β-actin. (**C**) Representative Western blots for Pgc-1α, Nrf1, and β-actin are shown at top; blot density obtained from densitometric scanning of Pgc-1α and Nrf1 normalized to β-actin is shown at the bottom. Data presented as means ± SEM of 6 rats in each group. * *p* < 0.05 vs. control.

We next examined the mRNA expression of Pgc-1α and Nrf-1, the two genes that regulate mitochondrial biogenesis and replication. Elevated maternal T levels significantly decreased Pgc1-α mRNA expression in the placenta (30% lower) compared with controls (Figure 4A). In addition, elevated T significantly decreased Nrf1 mRNA levels in placental tissue (33% lower) compared with controls (Figure 4B). Consistently, elevated T also suppressed Pgc1-α and Nrf1 protein levels in the placenta compared with controls (Figure 4C).

### 3.5. Mitochondrial Content and PGC-1α and NRF1 Gene Expression in Trophoblast Cells

**Figure 5:**
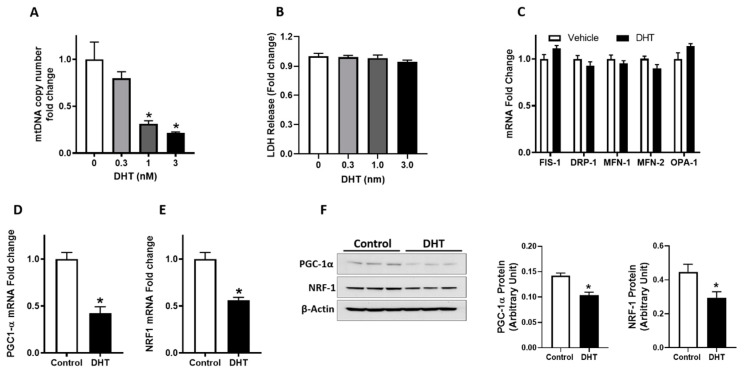
Mitochondrial copy number and expression of fission/fusion and biogenesis indicators. Trophoblasts cells were treated with vehicle (ethanol) or dihydrotestosterone (DHT) for 24 h. (**A**) Mitochondrial DNA copy number was quantified using qRT-PCR based analysis. (**B**) Cell viability after exposure to DHT was assessed using lactate dehydrogenase (LDH) cytotoxicity assay. The LDH levels were measured and expressed as the fold change compared to vehicle control. Real-time PCR was used to assess the relative mRNA expression levels of (**C**) fission/fusion genes (FIS-1, DRP-1, MFN-1, MFN-2, and OPA-1), and biogenesis indicators (**D**) PGC-1α and (**E**) NRF1, normalized to β-actin. (**F**) Representative Western blots for PGC-1α, NRF-1, and β-actin are shown at the left; blot density obtained from densitometric scanning of PGC-1α and NRF-1 normalized to β-actin is shown at the right data presented as means ± SEM of 4 biologically independent replicates. * *p* < 0.05 vs. control.

Based on the effects of hyperandrogenism on placental mitochondrial dysfunction, we next assessed the direct impact of T on mitochondrial dynamics in an in vitro cell culture model. DHT (a non-aromatizable form of T), at clinically relevant concentrations (0.3 to 3 nM), as observed in pregnant women [15], dose-dependently decreased mitochondrial DNA copy number in cultured trophoblast cells with a significant decrease occurring at 1 nM (−69%) and 3 nM (−79%) (Figure 5A *p* < 0.05, *n* = 4). DHT did not affect cell viability at the concentrations used in this study (Figure 5B).

To investigate the possible mechanism of T-induced mitochondrial dysfunction, we evaluated mitochondrial fission (FIS-1 and DRP-1) and fusion (MFN-1, MFN-2, and OPA-1) genes and biogenesis indicators. DHT, at 3 nM concentration, did not alter mitochondrial fission and fusion genes (Figure 5C) but induced a significant decrease in PGC-1α and NRF1 mRNA (Figure 5D,E) and protein expression (Figure 5F).

### 3.6. Cellular Bioenergetics in Trophoblast Cells

**Figure 6:**
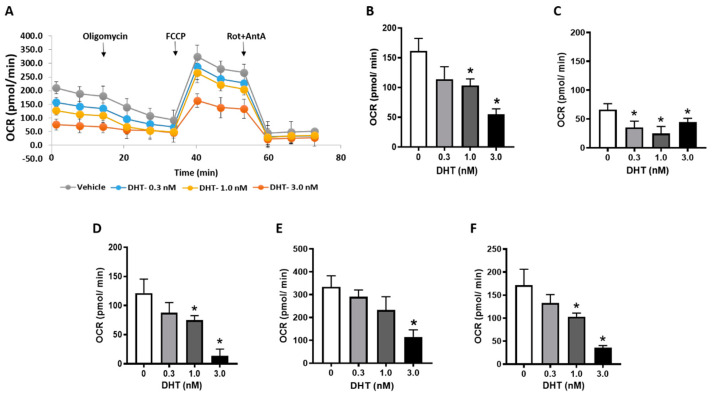
Bioenergetics profile of trophoblasts. Trophoblasts cells were treated with vehicle (ethanol) or dihydrotestosterone (DHT) for 24 h; mitochondrial respiratory parameters were measured using Seahorse. (**A**) Representative traces of mitochondrial respiration, (**B**) basal oxygen consumption rates (OCR), (**C**) proton leak, (**D**) ATP production-linked respiration (OCR after oligomycin administration), (**E**) maximal respiration (OCR after carbonyl cyanide 4-(trifluoromethoxy)phenylhydrazone (FCCP) administration) and (**F**) spare respiratory capacity (Difference between basal and maximal OCR). Data are presented as means ± SEM. The studies were done in duplicate from samples obtained from 4 biologically independent replicates. * *p* < 0.05 vs. vehicle control.

To investigate if DHT might interfere with mitochondrial function, we sought to examine DHT’s effects on cellular bioenergetics. The basal respiration and respiration after the sequential injection of compounds are depicted in Figure 6A. DHT dose-dependently impaired mitochondrial respiration as evidenced by a decreased basal respiration compared with vehicle controls (Figure 6B). This mitochondrial respiration is due to two components: oxygen consumption due to ATP synthesis and due to natural proton leak across the inner mitochondrial membrane. The addition of oligomycin (ATP synthase inhibitor) allows for these contributory components to be isolated. DHT-treated trophoblasts demonstrated significantly decreased proton leak at all concentrations tested (Figure 6C), but the ATP-linked respiration dose-dependently reduced with a greater effect observed at higher DHT concentrations (Figure 6D).

The addition of FCCP allows for an estimation of the maximum OCR. FCCP is an ionophore that directly transports protons across the inner mitochondrial membrane instead of via the ATP synthase proton channel. Hence, the addition of FCCP collapses Δψ_m_, leading to the rapid consumption of oxygen without the generation of ATP. DHT exposure produced a dose-dependent decrease in the maximum respiratory rate (Figure 6E). In parallel, the spare respiratory capacity also decreased with increasing DHT concentrations (Figure 6F).

## 4. Discussion

To the best of our knowledge, this is the first study to investigate the impact of elevated T levels on placental mitochondrial structure and function. Consistent with previous studies [23], elevated maternal T led to a decrease in placental and fetal weights. This study, for the first time, demonstrates that elevated T levels disrupt the structure and number of placental mitochondria with a parallel decrease in cellular bioenergetics in trophoblast cells. The reduced mitochondrial structure and function were associated with decreased PGC-1α and NRF-1 expression in the placenta and trophoblasts. Therefore, we suggest that elevated T plays an important role in causing mitochondrial dysfunction in the placenta, and the suppressed PGC-1α and NRF-1 expression and function might contribute to the reduced mitochondrial function and the associated placental and fetal growth restriction.

The placenta plays an integral role in the pathogenesis of PE. The mitochondrial dysfunction in the placenta has been widely accepted as a major physiological disturbance in PE. However, the underlying factor that impacts mitochondrial function is not known. Mitochondrial DNA mutations are commonly found in classical mitochondrial diseases; however, such mutations are rarely linked with PE [43,44]. This raises the possibility that acquired mitochondrial dysfunction, possibly due to exposure to stressors, such as the altered hormonal, cytokine, and antiangiogenic profile [31,45,46], could contribute to the placental mitochondrial dysfunction in PE. In this study, we hypothesized that elevated maternal T levels, as observed in PE, impacts mitochondrial dysfunction. To this end, we exposed pregnant rats to elevated maternal T levels. The T levels in normal pregnant women range between 100 and 150 ng/dl, and these are increased by 1.5- to 2.4-fold during PE [15]. Importantly, it is only T levels, and not other androgens that are increased in PE [15]. Our experimental studies mimicking the 2-fold increase in T levels similar to that in PE resulted in decreased placental growth and fetal growth restriction, which is consistent with previous findings [23].

The novel finding of this study is that the placental mitochondria of pregnant rats with elevated T had a structural disruption with condensed matrix and cristae. In addition, electron microscopic studies also revealed less abundant mitochondria, and consistently, the placental mitochondrial DNA copy number, which is a measure of mitochondrial content [47], was strongly decreased in the placenta of T rats. Similar mitochondrial structural damage and reduced numbers are observed in the placenta and cytotrophoblasts of PE women and in other placental insufficiency conditions [7,10,11,47,48]. Although the sex-dependent impacts of T on placental mitochondrial structure and function were not examined in this study, it will be an interesting aspect to examine in the future. Consistent with the disruption in the structure and number of mitochondria, elevated maternal T reduced the function of mitochondria, as evidenced by decreased ATP content and ATP/ADP ratio in the T placenta.

Previous studies report that elevated maternal T leads to impaired placental differentiation, reduced transplacental nutrient transport, and impaired spiral artery remodeling [17,21,22]. These experimental T-induced effects and the circumstantial evidence of elevated T present in PE and growth-restricted pregnancies suggest that maternal T-induced mitochondrial dysfunction could play a central role in causing placental insufficiency and fetal growth restriction. Indeed, studies have shown that placental mitochondrial dysfunction can lead to placental and fetal growth restriction [49].

To understand the possible underlying mechanisms for the decreased mitochondrial content in the T placentas, we focused on fission/fusion and PGC-1α-related intracellular machinery. Mitochondria undergo constant morphological change through cycles of fission and fusion, which are part of the mitochondrial response to adverse conditions [50]. The lack of changes in the expression of mitochondrial fission/fusion-related genes in our studies indicates that these processes are not likely altered in trophoblasts exposed to elevated androgen levels.

PGC-1α is a master regulator of mitochondrial oxidative metabolism [32]. Its expression reflects cellular energy needs, with conditions of increased energy demands inducing its expression [33]. PGC-1α stimulates the transcriptional activity of NRF1, which in turn, regulates mitochondrial DNA transcription and replication [34,35]. We found that elevated maternal T significantly decreased Pgc-1α and Nrf1 expression in the placenta. In line with this observation, reduced placental Pgc-1α expression is noted in rats subjected to reduced uterine perfusion pressure (an animal model of PE) [51]. Importantly, a reduced expression of PGC-1α and NRF1 is also observed in the placenta of PE and growth-restricted pregnancies, correlating with the reduced mitochondrial content in them [47,52]. This raises the possibility that a T-induced decrease in Pgc-1α and Nrf1 expression may contribute to the downstream effect of reduced mitochondrial content. This notion is supported by DHT decreasing PGC-1α and NRF1 mRNA expression along with a parallel reduction in mitochondrial content in cultured trophoblasts without negatively affecting cell viability. Furthermore, these in vitro findings in trophoblasts indicate that androgens can directly downregulate the transcription of these mitochondrial regulators independent of any other in vivo endogenous factors. Although our studies implicate that T induces a decrease in PGC-1α and its downstream NRF1 expression, the underlying molecular mechanisms are still unclear. PGC-1α and NRF1 are downregulated at the mRNA level, suggesting that T may induce the decrease at the level of transcription. Analysis of the PGC-1α and NRF1 promoter shows no presence of putative androgen response elements. It is possible that T could downregulate PGC-1α transcription from alternative response elements such as the glucocorticoid response element [53]. In addition, the proximal PGC-1α promoter contains a typical PPAR response element [54], and PPARγ positively stimulates PGC-1α transcription [55]. T could suppress PGC-1α transcription by inhibiting PPARγ expression as in ovarian granulosa cells [56]. The exact mechanism by which androgens regulate PGC-1α and NRF1 remains to be elucidated. Intriguingly, recent studies show that T upregulates Pgc-1α and Nrf1 in granulosa cells and skeletal muscles, respectively [57,58]. It would be interesting to examine if the T-induced decrease in PGC-1α and NRF1 in the placenta is a tissue-specific or pregnancy-specific effect.

The mitochondrial respiration was decreased in DHT-exposed trophoblasts compared to vehicle controls. In particular, decreased basal respiration, ATP-linked respiration, and maximal respiration were observed. This is indicative of reduced electron flow through the respiratory chain, which is coupled to reduced oxidative phosphorylation. In addition, these findings could also be likely due to the reduction in the number of mitochondria per cell. Exposure to DHT resulted in a more precipitous decrease in the reserve capacity trophoblasts. This is significant, since reduced reserve capacity is linked to several diseases, such as aging [59] and neurodegenerative disorders [60]. The depletion of reserve capacity renders the cell unable to meet any additional ATP demand, which usually occurs under conditions of decreased mitochondrial content or electron transport chain inhibition [60]. This reduction in mitochondrial respiration in the DHT exposed trophoblasts is consistent with clinical reports of reduced mitochondrial content and inhibited electron transport chain activities in the placenta of PE and growth-restricted patients [10,47,61]. It is unclear whether androgens, in addition to decreasing mitochondrial content, also diminish the enzymatic activities of the respiratory chain complexes, which should be investigated in the future.

## 5. Conclusions

This study identified that elevated T levels, as observed in PE pregnancies, induced disruption in structure and the number of placental mitochondria with a parallel decrease in placental and fetal weights. In addition, elevated T impaired cellular bioenergetics by decreasing basal, ATP-linked, and maximal respiration in trophoblast cells. In addition, T decreased PGC-1α and NRF1 expression in the placenta and trophoblasts. The results demonstrate a novel role of T in the perturbations of placental mitochondrial function. The suppressed PGC-1α and NRF1 expression and the resultant decrease in placental mitochondrial content, and bioenergetics caused by elevated T levels suggest new insights of molecular mechanisms linking PE to feto-placental growth restriction. Strategies that target excessive androgen action in the placenta could have important therapeutic potential in the treatment of pregnancies complicated by PE and fetal growth restriction.

## Figures and Tables

**Table 1:** Quantitative real-time PCR primer sequence.

Gene	Forward	Reverse	Species
Pgc-1α	GAGTCTGAAAGGGCCAAGC	GTAAATCACACGGCGCTCTT	Rat
Nrf1	GGCGCAGCACCTTTGGAGAATGTG	CATCGATGGTGAGAGGGGGCAGTTC	Rat
FIS-1	TACGTCCGCGGGTTGCT	CCAGTTCCTTGGCCTGGTT	Human
DRP-1	TGGGCGCCGACATCA	GCTCTGCGTTCCCACTACGA	Human
MFN-1	GGCATCTGTGGCCGAGTT	ATTATGCTAAGTCTCCGCTCCAA	Human
MFN-2	GCTCGGAGGCACATGAAAGT	ATCACGGTGCTCTTCCCATT	Human
OPA-1	GGCTCTGCAGGCTCGTCTCAAGG	TTCCGCCAGTTGAACGCGTTTACC	Human
PGC-1α	CGCAGTCACAACACTTACAAGC	GGGGTCATTTGGTGACTCTG	Human
NRF1	GGCACTGTCTCACTTATCCAGGTT	CAGCCACGGCAGAATAATTCA	Human
